# Can social enterprises aid sustainable development? Evidence from multi-stage investigations

**DOI:** 10.1371/journal.pone.0281273

**Published:** 2023-02-13

**Authors:** Sanchita Bansal, Isha Garg, László Vasa

**Affiliations:** 1 University School Management Studies, Guru Gobind Singh Indraprastha University, Dwarka, New Delhi, India; 2 Faculty of Economics, Széchenyi István University, Győr, Hungary; Wroclaw University of Economics and Business: Uniwersytet Ekonomiczny we Wroclawiu, POLAND

## Abstract

**Background:**

Social enterprises must balance between profitability and sustainability. The impetus on sustainability grew further after the adaption of the SDG agenda by the United Nations (UN).

**Objective:**

This paper examines the role of social enterprises in helping attain sustainable development goals in India.

**Research design:**

This multi-stage paper comprises three studies directed at scale development, scale refinement, pre-testing, and construct validity. The scale development stage is conducted through a combination of extensive literature review and focused group discussions. Expert discussions and item-reduction techniques have been used in the second stage aimed at scale refinement and pre-testing. The third stage of testing construct validity is carried out through Partial Least Square–Structural Equation Modelling (PLS-SEM).

**Results:**

The findings suggest that all the competencies of social enterprises identified in the study i.e. social mission, collaborative networks, innovation, financial viability and level of scalability significantly impact sustainable development.

**Conclusion:**

Because the goals of social enterprises and the Sustainable Development Goals (SDGs) are mutually reinforcing and complementary, the study suggests that social enterprises have a lot of potential for achieving the SDGs. The study also suggests future research directions and policy implications that can be replicated in other countries.

## Introduction

Seven years have passed since the Sustainable Development Agenda 2030 was adopted by the United Nations (UN) in September 2015. With eight years left to achieve the SDGs set out in the agenda, countries assess their progress and determine their next course of action [1]. SDGs are comprehensive and focus on the five Ps–people, planet, prosperity, peace, and partnership [2]. These ambitious and aspirational SDGs necessitate a significant rethinking of global development processes. They also demand that significant resources be devoted and spent in priority areas designated in each member state’s framework of Goals and Targets [3]. Initially, governments worldwide had been known to carry out the SDG agenda and deal with the global challenges; however, businesses have now emerged as alternatives in attaining these goals [4]. As a result, such businesses are given top priority in national policies. The government has expressed a desire to develop these businesses as an integral part of the country’s development challenges [5]. To address long-term development challenges, the United Nations, in particular, urged all businesses to use their creativity and innovation [6,7]. Furthermore, Littlewood & Holt [8]; Rahdari et al. [9] highlight that businesses with a social purpose or social enterprises have an important role to play in achieving inclusive growth and attaining these goals. Therefore, the importance of social enterprises has grown even more since the implementation of the 2030 Agenda [10]. They can solve the problem by altering the system rather than entrusting societal needs to the government or business sectors [11].

Yunus et al. [12] define social enterprises as–

*"[While] its primary purpose is to serve society*, *a social business has products, services, customers, markets, expenses, and revenues like a ’regular’ enterprise. It is a no-loss, no-dividend, self-sustaining company that repays its owners’ investments."*

Social enterprises act as a hybrid organization between a commercial enterprise and a non-profit organization. The primary distinction between social enterprise and traditional commercial enterprises is found in their primary goals. Unlike commercial enterprises, social enterprises prioritize the creation of social value over economic value [13,14]. On the other hand, the ability of social enterprises to generate income distinguishes them from non-profit organizations [8,15].

South Asia’s problems are unique and distinct from those of the developed world [16]. It presents a different perspective, and hence, to address such challenges from various angles, there is a need to discuss holistic development. India is being chosen as the context for this research because it is one of the emerging economies experiencing a significant increase in social enterprises and is regarded as a potential environment to launch a social enterprise [17]. India is the world’s second-most populous country, with a diversified economic structure and a variety of political issues, including bureaucracy. As a result, the objective of social welfare cannot be realized just by the government; it requires support from the general public and entrepreneurs [18].

**Fig 1:**
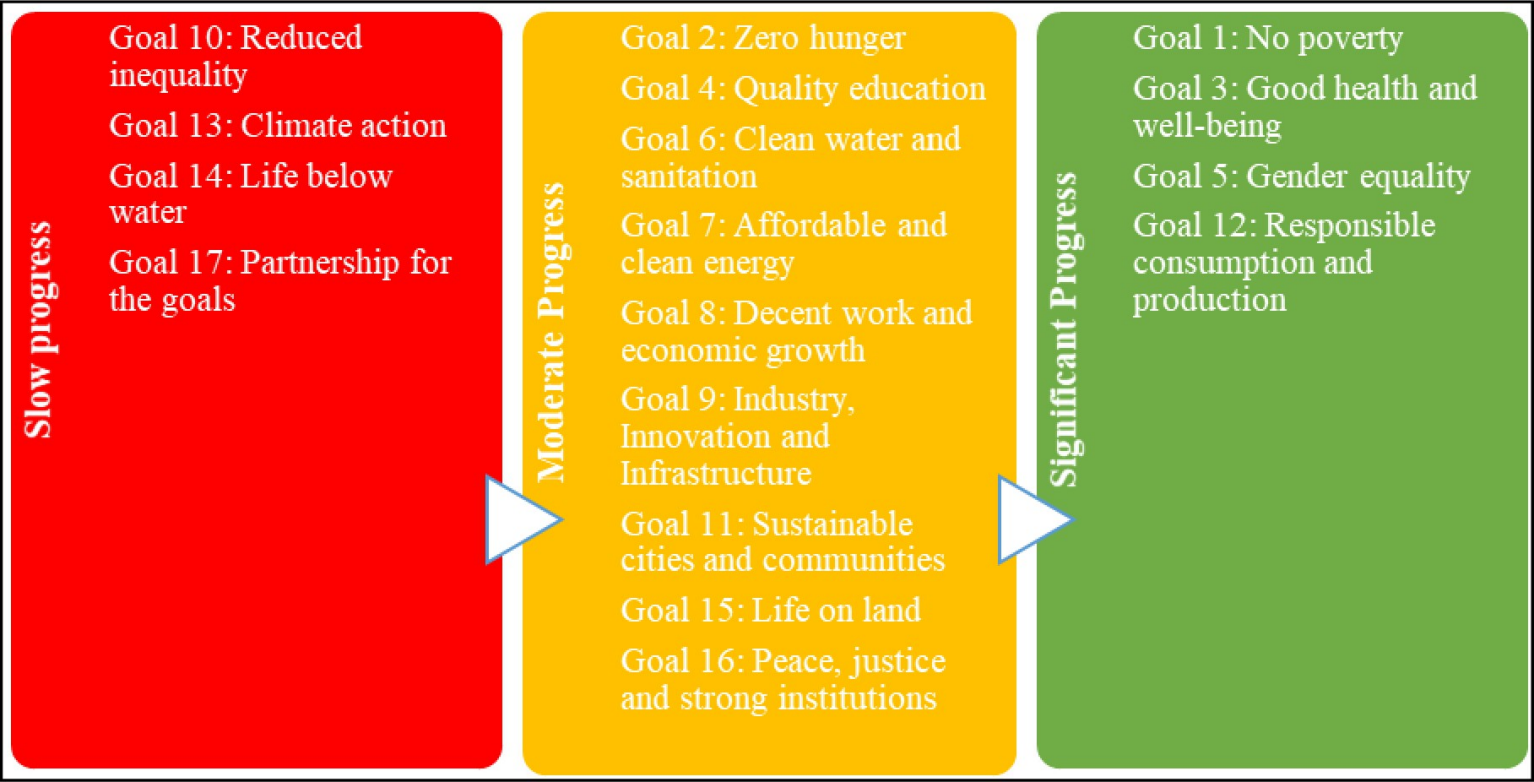
Progress towards 17 SDGs in India. (**Source**: Authors’ Contribution).

The Government of India is taking several initiatives to advance these goals through various policies, programs, and schemes [19,20]. Ministry of Statistics and Program Implementation [21] summarizes the progress made thus far in achieving the SDGs. The report also includes data-driven evidence of progress toward the SDGs and their associated targets. It also assists policymakers in determining which thrust areas require intervention. According to the report while significant progress has been made in some areas, challenges remain in others. Fig 1 highlights the progress made towards these goals in India.

This multi-stage paper includes three stages: Stage 1—Extant Review of Literature and Scale Development; Stage 2—Scale Refinement; and Stage 3—Measurement Model and Validity. Stage 1 presents an integrative review of the literature. The extant literature review is based on 52 papers from Web of Science (WoS) and 43 additional papers from various databases to establish a baseline for the definition of the constructs and item generation. A structured questionnaire is framed using the review. Along with the review of the literature, a comprehensive qualitative method, that is, focus group discussion, is used with six experts in the field to develop the theoretical framework presented in the study. Stage 2 involves refining the questionnaire, which involves establishing the validity and reliability of the questionnaire. The validity is established by conducting discussions with experts, and for reliability, the study follows the criteria given by [22]. Statistical tests have been conducted on a pilot study on a sample of 50. Stage 3 involves assessing the structural as well as measurement model using PLS-SEM.

The review of literature and focus group discussions led us to the scale developed for the study. PLS-SEM is used to analyze the questionnaire results. The study includes the examination of measurement as well as the structural model. The measurement model is evaluated using indicator reliability, internal consistency, convergent validity, and discriminant validity. Two indicators have extremely low values for indicator reliability, so they are omitted from the analysis. The paper examined VIF values, coefficients of determination, path coefficients, and predictive relevance to evaluate the structural model.

The relationship between social enterprises and the SDGs, and more significantly how social enterprises might help accomplish the SDGs, has received little attention in the existing social enterprise literature. Social enterprises typically arise from a local setting in which communities or social groups establish a business initiative that benefits their stakeholders through generating social, economic, and environmental benefits for them. Social entrepreneurs are crucial for the bottom of the pyramid as they develop low-cost products and services that they can use. At the same time, these enterprises address issues including unemployment, social exclusion, marginalization, and a lack of market or financial resources. The potential of social enterprises to alleviate poverty through market mechanisms may also give a long-term solution to this problem [23]. The majority of past research on the relationship between social enterprises and sustainable development has concentrated on developed countries [24]. In developing countries, there are less papers on the relationship. Despite minimal study in developing countries, the concept has gained traction since the 1990s [25]. Because details concerning social concerns in developing nations and the strategies employed to tackle them are scarce, Murthy et al. [26] underline the need of exploring the idea in developing countries. Furthermore, Lee & Seo [27] highlight that many studies have proposed different models for social enterprises, however their study is restricted in that it just provides a framework without further empirical investigation. Therefore, Certo & Miller [28] underline the necessity of empirical studies that might contribute to a better understanding of social entrepreneurship.

Therefore, the study contributes significantly to the literature in four ways. First, the study emphasizes the importance of social enterprises in achieving economic, social, and environmental sustainability to better global society. The paper proposes a conceptual framework that integrates social enterprises and sustainable development. Second, the study empirically tests how social enterprises work toward achieving sustainable development and eventually discusses their contribution toward SDGs. Third, the study has been carried out in India, a developing country, to replicate the findings in other countries. Fourth, the study used an integrative review to look at the current state of the literature, identify gaps in the existing literature, and suggest areas for future research in the field.

The rest of the paper is organized as follows. Section 2 presents the methodology. Section 3 presents Stage 1 (Extant Review of Literature and Scale Development). Section 4 presents Stage 2 (Scale Refinement). Section 5 presents Stage 3 (Measurement Model and Validity). Section 6 provides a discussion. Section 7 presents the conclusion. Section 8 presents implications of the study and limitations.

## Material and methods

The objective of this multi-stage paper is to determine the impact of social enterprises on sustainable development. It includes three studies–Extant Literature Review and Scale Development; Scale Refinement; and Measurement Model and Validity. This section describes the research methodology used in the study.

Table 1 presents the aims of the studies conducted. Stage 1, involving a bibliometric analysis and focus group discussion, aims to develop the theoretical framework and scale for the study. The first draft of the questionnaire is prepared with 36 Likert-scale statements. Stage 2 aims at refinement and pre-testing of the questionnaire, which is carried out using expert discussions and reliability and validity tests. Five statements are dropped, and the questionnaire is left with 31 statements. Finally, Stage 3 analyzed the results obtained from the questionnaire using PLS-SEM. After the analysis, two statements are dropped, leaving the questionnaire with 29 statements.

**Table 1:** Studies conducted.

S. No.	Aim	Activity	Method	Outcome
**1.**	Scale Development	1. Statement generation	Bibliometric analysis (52 papers), Literature review (52 + 43 papers) and Focused Group Discussions (n = 6)	Theoretical Framework, Questionnaire with 36 statements
**2.**	Scale refinement and Pre-testing	1. Scale refinement 2. Pre-testing	Expert discussion (n = 4), Mean, Item-item correlation and Cronbach’s alpha	Statements reduced to 31
**3.**	Construct Validity	Discriminant Validity	PLS-SEM	Statements reduced to 29

Data collection is divided into three stages. The first stage involves data collection for the bibliometric review [29]. Data was collected in the first stage using the Web of Science database [30]. Only peer-reviewed English literature papers were chosen, resulting in 52 papers. The data was then analyzed using the Bibliometrix R package software [31,32].

The data collection for the theoretical framework is the second stage. 43 papers were added to the existing data from various databases such as Scopus, Science Direct, and Google Scholar to prepare the theoretical framework and scale development. Following the literature review, focus group discussions are used for the theoretical framework. Five characteristics of social enterprises have been identified for achieving sustainable development. Also, the first draft of the questionnaire was developed with 36 Likert-scale items.

The third stage of data collection involves data for the structured questionnaire for empirical analysis. Data was collected via an online questionnaire, developed and distributed to social entrepreneurs through "Google Docs" [33,34]. By collecting data from a large geographical area, web-based data collection saves time and money [35], and it was a specifically relevant tool during the global pandemic.

Since the database for social entrepreneurs is not publically available, we contacted Central and State government agencies for the same. The study uses the purposive sampling technique. Purposive sampling involves researchers selecting individuals based on their unique traits, such as their attitudes, experiences, or perceptions; new participants are sought to question existing trends. Purposive sampling looks for those who have a "strong interest in and the ability for analytical thinking and figuring out how things function" [36]. The study collected data from social enterprises that are supported by a government body. The government is promoting schemes to support research and entrepreneurial skills among youth through various programs. It is also supporting youth who are coming forward to develop sustainable solutions for the betterment of society at large and through these initiating their entrepreneurship journey.

The questionnaire was sent to 2000 social entrepreneurs for which data we received. The survey received 350 responses. The response rate was 17.5 percent. Following data collection, data cleaning was performed to remove outliers, incomplete questionnaires, and duplicates. As a result, 45 of them were removed. There were 305 final questionnaires. The final data was analyzed using PLS-SEM.

### Stage 1 –extant review of literature and scale development

This section presents a review of the extant literature. This literature review aims to gain a thorough understanding of social enterprises’ impact on sustainable development. Hence, the authors conducted an integrative review to identify various aspects and present the state of knowledge in social enterprises and sustainable development literature. The information for this study came from the Thomson Reuters Web of Science (WoS) Index. The search was limited to English-language peer-reviewed journal articles and covered all available research years up to and including 2021. This refinement resulted in 52 research articles being exported as a ’.txt’ file, complete with title, abstracts, keywords, authors, publications, citations, references, countries, affiliations, and other information. Following that, the data was analyzed using the Bibliometrix R package software [31,32].

This section is divided into two sub-sections. The first sub-section presents the results and findings of an integrative review of the selected research articles. The second sub-section presents the conceptual framework proposed for the existing study.

#### A brief integrative review

This section examines the role of social enterprises in achieving long-term development using bibliometric analysis.

#### Publication trend and citation structure

**Fig 2:**
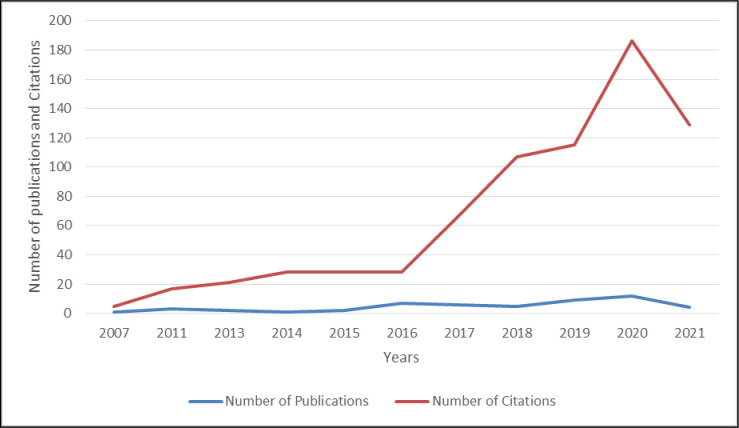
Publication trend and citation structure. (**Source**: Authors’ Contribution).

Fig 2 presents the publication trend and citation structure on the role of social enterprises in attaining sustainable development. The figure reveals a rising trend, indicating that researchers are becoming more interested in this field. The year 2020 has the highest number of publications and citations.

#### Thematic map and data structure

**Fig 3:**
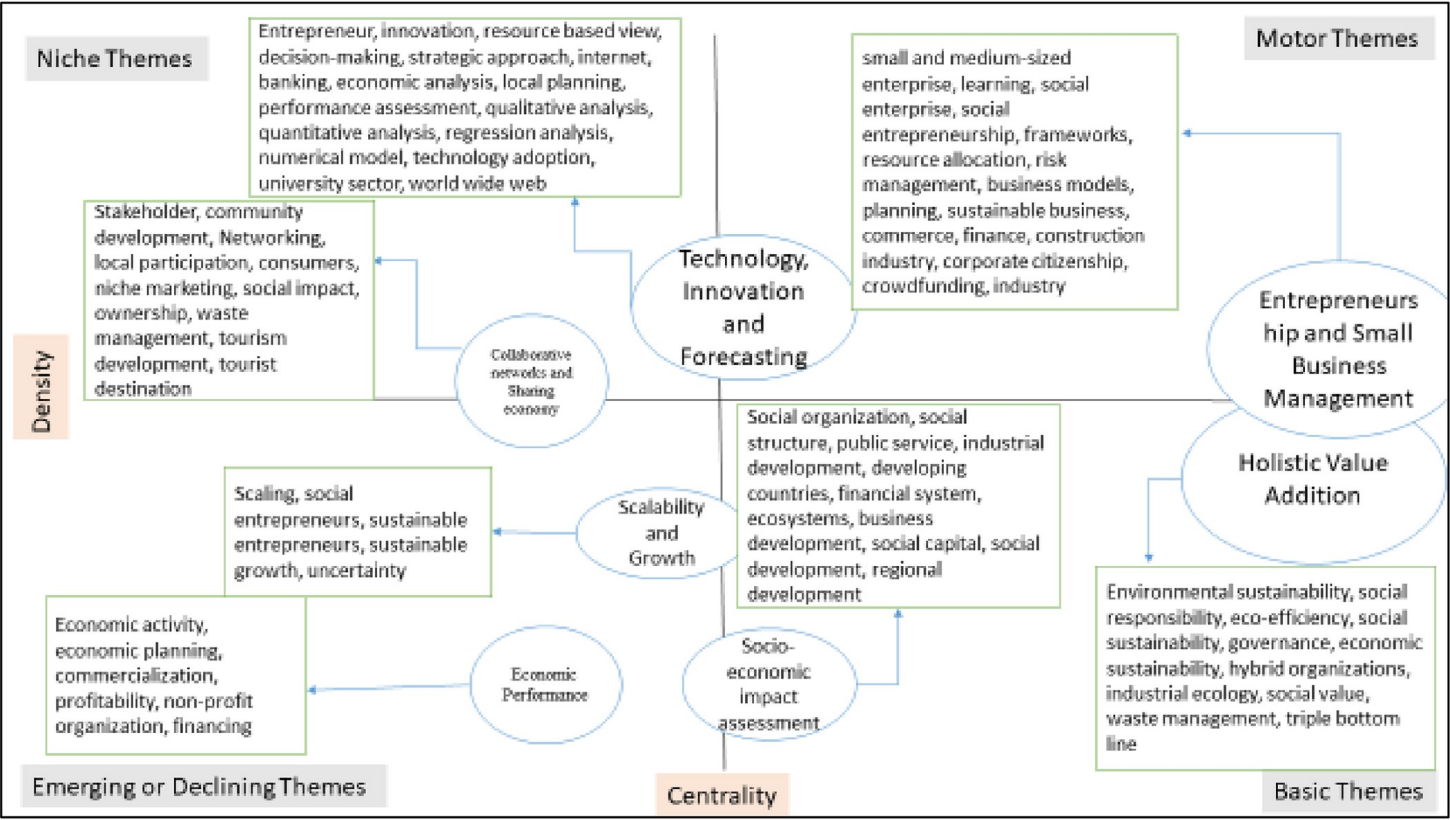
Thematic map. (**Source**: Authors’ Contribution).

The themes represented in the thematic map (Fig 3) are categorized according to their centrality and density [37]. The degree of interaction between the themes is measured by centrality, while the strength of internal ties within a theme is measured by density [32,38]. For example—the themes appearing in the fourth quadrant with high centrality and low density imply weaker internal ties, indicating the need for more research into the sub-themes in the future. The number of publications associated with each of these themes is indicated by the size of the theme, indicating that these are focused themes.

Motor themes have two themes, namely Entrepreneurship and Small Business Management; Technology, Innovation, and Forecasting. As can be seen from the figure, the theme Entrepreneurship and Small Business Management has the highest number of publications. It includes keywords such as small and medium-sized enterprise, learning, social enterprise, social entrepreneurship, and frameworks. Although within the field of entrepreneurship, social entrepreneurship has emerged as a sub-discipline [28], the concept of social entrepreneurship and its components are not well understood or agreed upon [8,24,39]. The existing studies present various entrepreneurship frameworks [27,40], but their research is limited in that it only presents a framework and does not go into further empirical detail. The second theme, Technology, Innovation, and Forecasting, has keywords including entrepreneur, innovation, resource-based view, decision-making, strategic approach, internet, banking, and economic analysis. Social entrepreneurs can act without being constrained by available resources [41]. Organizations use frugal innovation to provide innovative products or services to underprivileged customers in economically constrained environments [42–45]. When looking at social entrepreneurial organizations from the resource-based view, it is clear that their resources and talents are crucial to social innovation and the growth of entrepreneurial initiatives [46,47]. Competitive advantage is translated into sustainability by effectively exploiting packaged valuable resources [48].

Niche themes have one theme, namely Collaborative Networks and Sharing Economy. It includes keywords such as stakeholder, community development, networking, local participation, consumers, and niche marketing. Social enterprises (SEs) create a naturally conscious environment, blurring the line between society and business [49]. Social enterprises benefit everyone and significantly impact the communities where they operate [50]. As a result, research is required to assess the impact of their activities on community development and other stakeholders. Cooperation between enterprises can develop their core professionalism, enhance their brand image, and embrace the effects of synergy, allowing them to solve social problems [49,51]. Several studies have been conducted to see how managers use commercial and social marketing activities to achieve sustainable goals. The findings revealed that social companies could attract target consumers by engaging in niche marketing [52]. The new sharing economy paradigm incorporates social entrepreneurs’ importance into mainstream sustainable development literature [9].

Basic themes include two themes, namely–Holistic Value Addition [53] and Socioeconomic impact assessment. The theme holistic value addition includes keywords such as environmental sustainability, social responsibility, eco-efficiency, social sustainability, governance, developing countries, and economic sustainability. Holistic value includes social, economic, and environmental sustainability. Many businesses are making investments in environmental sustainability [54]. Almost all businesses aim to provide green products and services using environmentally friendly materials and processes and/or disclosing their social and environmental performance due to greater public awareness [49,55]. Given the urgency of social and environmental issues, it is critical to comprehend the political dynamics that encourage sustainable development [56]. Social entrepreneurs have arisen as agents who use entrepreneurial means to address environmental issues [57]. Proper policy implementation and governance are required to not harm the future by the present to promote sustainable development. Only through high governance standards at all levels can sustainable development be achieved [58]. Socioeconomic impact assessment includes social organization, social structure, public service, industrial development, developing countries, financial system, ecosystems, and business development. Social entrepreneurs are mindful of the expectations and values of their investors, which include anyone who helps them with money, time, or skill. They strive to give tangible social advantages to their beneficiaries and communities, as well as a compelling (social and/or financial) return on investment to their investors [41]. These businesses prioritize their social, ethical, and environmental missions over profit. Dees [41] also argues that the primary goal of a social entrepreneur is to create superior social value.

The emerging or declining themes involves two themes, Scalability and Growth; Economic performance. The theme scalability and growth includes keywords such as scaling, social entrepreneurs, sustainable entrepreneurs, sustainable growth and uncertainty. To accomplish growth and secure the long-term viability of the social enterprise, the social entrepreneur must expand their business and manage resources with both a commercial and a social purpose, enhancing the business’s long-term sustainability. As a result of business expansion, the internal context of the social enterprise changes, prompting a change in the role of the social entrepreneur. This may entail fundamental outsourcing responsibilities or hiring outside assistance to run and grow the business [59]. However, because the growth of social enterprises has gotten little attention in the literature, there is a need to delve more into the field. The theme Economic performance includes keywords such as economic activity, economic planning, commercialization, profitability, non-profit organization and financing. While attempting to achieve their social goal, social businesses must recover all of their costs to be self-sustaining. Their owners have no intention of making profits (there are no dividends), but they are entitled to their money back if they so desire. Surpluses generated by the social business are reinvested in the business rather than being passed on to investors and thus eventually passed on to the target group of beneficiaries [12].

**Fig 4:**
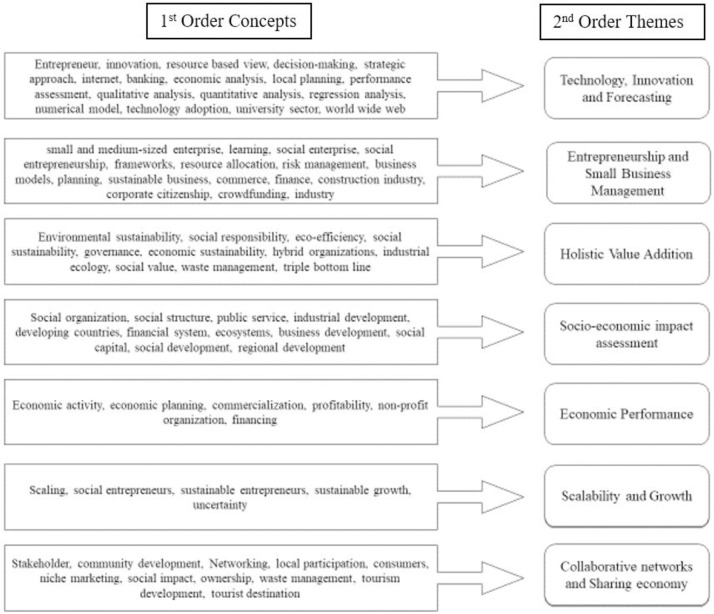
Data structure. (**Source**: Authors’ Contribution).

**Fig 5:**
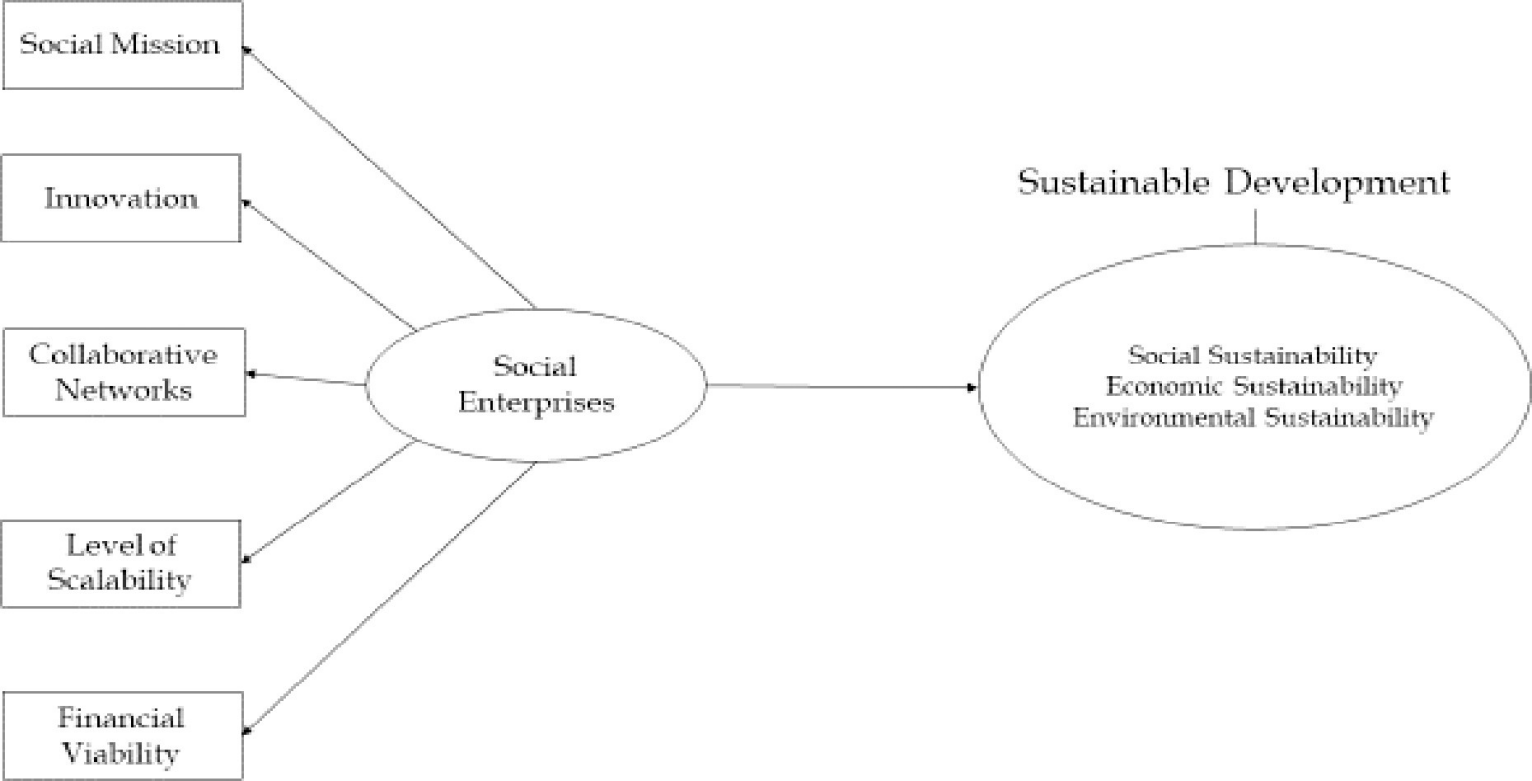
Theoretical framework. (**Source**: Authors’ Contribution).

The Data structure shows how the keywords form the themes. The thematic map (Fig 3) and the Data structure (Fig 4) developed from the literature have supported the development of the theoretical framework (Fig 5).

*Theoretical framework*. The 52 articles retrieved for bibliometric analysis were then combined with 43 additional articles retrieved from Scopus, Science Direct, and Google Scholar databases. In addition to the review of literature, focus group discussions are conducted to develop the theoretical framework (Fig 5) for the study. It has assisted in identifying five social enterprise competencies and three dimensions of sustainable development. The five competencies of social enterprises are social mission, collaborative networks, innovation, financial viability and level of scalability. The three pillars of sustainable development are social, economic and environmental [46,60].

#### Social mission

For social entrepreneurs, the social objective is apparent and central. This has a clear impact on how social entrepreneurs see and assess possibilities. The primary criterion becomes mission-related effect rather than income creation [41,61]. By their very nature, social enterprises are balancing acts that must reconcile two often opposing goals: achieving a social mission and achieving financial success in the marketplace. This is true regardless of the organization’s structure or location; its owners are committed to a social mission [62].

H_1_: Social mission positively impacts sustainable development

H_1a:_ Social mission positively impacts social sustainability

H_1b:_ Social mission positively impacts economic sustainability

H_1c:_ Social mission positively impacts environmental sustainability

#### Innovation

Zahra et al. [63] define social entrepreneurship as "*the activities and processes undertaken to discover*, *define*, *and exploit opportunities in order to enhance social wealth by creating new ventures or managing existing organizations in an innovative manner*". A common belief in the literature on social enterprises, which is deeply rooted in the entrepreneurship tradition, is that they are inherently innovative [64]. In each of the 17 SDGs’ focus areas, social entrepreneurs are problem solvers [65]. Also, enterprises that succeed in today’s competitive environment tend to invest in technology to obtain a competitive advantage. Adoption of technology is frequently based on the CEO’s or other top executives’ wishes and aspirations [66]. Given the magnitude of the world’s current societal problems, social enterprises are required for their innovative solutions [65].

H_2_: Innovation positively impacts sustainable development

H_2a:_ Innovation positively impacts social sustainability

H_2b:_ Innovation positively impacts economic sustainability

H_2c:_ Innovation positively impacts environmental sustainability

#### Collaborative networks

Individuals and organizations connected through collaborative networks share their ideas and resources with one another [67]. Enterprises are no longer self-sufficient and can no longer exist on their own. To survive and grow, they require resources and information. Social networks act as a catalyst for social enterprises’ long-term success. Entrepreneurs use networks to obtain information, resources, assistance, opportunities, advice, and market knowledge [34]. The synchronization of business goals with human goals will holistically redefine the business model, generating value for all stakeholders. The stakeholders for an enterprise include—individuals (shareholders, other investors, bankers, managers, employees, suppliers, customers, and competitors), families of individual stakeholders, society (government, activists, and communities), and nature [68]. The goal of social enterprises and their solution-oriented work is to benefit all stakeholders [65].

H_3_: Collaborative Networks positively impact sustainable development

H_3a:_ Collaborative Networks positively impact social sustainability

H_3b:_ Collaborative Networks positively impact economic sustainability

H_3c:_ Collaborative Networks positively impact environmental sustainability

#### Level of scalability

The ability to successfully replicate the business model in other locations and continue to generate more benefits is referred to as scalability in social enterprises [69]. The social enterprise originated as part of a small community center to offer skills training and boost employment prospects for local inhabitants and social facilities such as a public house and a network of community wardens to maintain the estate’s safety. The success of these initiatives fueled the company’s rapid growth and increased demand for services, which were then offered throughout a broader geographic area [14].

H_4_: Level of Scalability positively impacts sustainable development

H_4a:_ Level of Scalability positively impacts social sustainability

H_4b:_ Level of Scalability positively impacts economic sustainability

H_4c:_ Level of Scalability positively impacts environmental sustainability

#### Financial viability

Any social enterprise that does not guarantee financial performance will struggle to thrive. In general, financial performance should be the outcome of operational excellence, and economic prosperity should be a prerequisite for continued social contribution. As a result, a social enterprise can only reinvest its profits back into the firm or directly into the community if its sustainability is assured. Business operations’ performance should be evaluated to ensure social activities’ long-term survival [27].

H_5_: Financial Viability positively impact sustainable development

H_5a:_ Financial Viability positively impact social sustainability

H_5b:_ Financial Viability positively impact economic sustainability

H_5c:_ Financial Viability positively impact environmental sustainability

#### Sustainable development

Uneven development, population growth and poverty are all linked to many critical survival issues. They all put unprecedented strains on the planet’s lands, waters, forests, and other natural resources, particularly in developing countries. A waste of opportunities and resources is the downward spiral of poverty and environmental degradation. It is a waste of human resources in particular [70]. Now is the time for a new era of economic growth, one that is both forceful, socially and environmentally sustainable [71]. The concept of sustainable development, which has become central to the environmental debate, entails integrating environmental thinking into every aspect of social, political, and economic activity. Businesses are now developing new "win-win-win" strategies that benefit the company, its customers, and the environment all at the same time [72]. Therefore, the three pillars of sustainable development are social, economic, and environmental [73].

Social enterprises facilitate social change by addressing critical social needs and social values in order to develop an economic value for any economy [74,75]. Social enterprises are often created in response to unmet community needs. They utilize resources that are either unused or deemed useless by organizations, improving their use of such resources as necessary to meet their goals [14]. Various fields have seen an increase in the number of social enterprises such as healthcare, agriculture, education, energy, water and sanitation, affordable housing, livelihoods promotion and financial inclusion [76].

Using the above (3.1 and 3.2), the first draft of the questionnaire was prepared with 36 questions in the form of a 5-point Likert scale. The Likert scale has been used [33,77] to measure respondents’ agreement with the statements on a 5-point scale from ’strongly agree’ to ’strongly disagree’ [78,79]. The above discussion and the theoretical framework lead us to the following hypotheses:

### Stage 2—scale refinement

Scale refinement and validation has been established using validity and reliability tests using the criteria given by [22]. To test the validity of the questionnaire, we conducted expert discussions with four experts in the field from various parts of the world. The statements were reworded and rewritten in response to the experts’ suggestions.

The reliability of the questionnaire was tested statistically on a pilot study (a sample of 50 respondents). Reliability has been established using the following statistics [22]:

#### Mean

Items with means less than 2 or greater than 4 should be rejected, according to [80]. All the values were in the range of 2–4. Therefore, we did not drop any items after this test.

#### Item-total correlations

The item-total correlation should not be less than 0.25 [81]. We conducted an item-total correlation test in SPSS, removed the item with the lowest value of less than 0.25, and reran the test. We continued with the procedure until all the values are greater than 0.25. Five items were not fulfilling the criteria; therefore, we dropped those items one by one. The total remaining items were 31.

#### Cronbach’s alpha

The value of the alpha coefficient yielded is 0.92, which exceeds 0.70, as suggested by [82]. According to [83], internal consistency of 0.90 and above is excellent, 0.70–0.90 is good, 0.60–0.70 is acceptable, 0.50–0.60 is poor and below 0.50 is unacceptable.

#### Stage 3 –measurement model and validity

This section presents the data analysis carried out using Partial Least Square–Structural Equation Modelling (PLS-SEM). We used reflective model assessment guidelines for analysis because all of the constructs and the proposed model are reflective. Researchers should explore using PLS-SEM in scenarios where theory is less developed. PLS-SEM uses available data to estimate the path relationships in the model to minimize the error terms of the endogenous constructs. In other words, PLS-SEM estimates coefficients that maximize the R^2^ values of the (target) endogenous constructs. This feature achieves the prediction objective of PLS-SEM. PLS-SEM works efficiently with small sample sizes. It can therefore be applied in a wide variety of research situations. When applying PLS-SEM, researchers benefit from high efficiency in parameter estimation, which is manifested in the method’s greater statistical power than that of CB-SEM. Greater statistical power means that PLS-SEM is more likely to render a specific relationship significant when it is, in fact, significant in the population [84–86].

The analysis involves assessing the measurement model as well as the structural model. Researchers must evaluate the structural model if the measurement models match the required criteria. Table 2 shows the evaluation criteria of both measurement and structural models.

**Table 2:** Evaluation criteria of measurement and structural model.

Evaluation of the Measurement Model	Evaluation of the Structural Model
• Indicator reliability • Internal consistency • Convergent validity • Discriminant validity	• Variance Inflation Factor (VIF) • Coefficients of determination • Path coefficients • Predictive relevance • Effect Size

#### Assessing measurement model

*Indicator reliability*. The first step in assessing the measurement model is to examine the indicator loadings [84]. Higher loadings indicate that associated indicators share a lot of similarities, which the construct captures. Therefore, loadings greater than 0.708 are recommended [85]. Weaker outer loadings are frequently observed in social science studies, especially when using newly developed scales [87]. In general, indicators with outer loadings between 0.40 and 0.70 should only be removed from the scale if doing so increases the composite reliability and content validity. Some indicators with lower outer loadings are kept because of their contribution to content validity. However, indicators with very low outer loadings (below 0.40) should be eliminated off the scale at all times [85].

Table 3 represents the outer loadings. The table shows that two indicators (that is, FV5 and LS2) have loadings less than 0.4; therefore, these indicators have been removed. All the other indicators are falling within the acceptable range.

**Table 3:** Indicator loadings.

	Social mission	Innovation	Collaborative networks	Level of Scalability	Financial Viability	Social sustainability	Economic sustainability	Environmental sustainability
**SM1**	0.64							
**SM2**	0.76							
**SM3**	0.6							
**SM4**	0.79							
**I1**		0.64						
**I2**		0.73						
**I3**		0.65						
**I4**		0.83						
**CN1**			0.61					
**CN2**			0.73					
**CN3**			0.74					
**CN4**			0.78					
**LS1**				0.73				
**LS2**				0.22				
**LS3**				0.78				
**LS4**				0.79				
**FV1**					0.57			
**FV2**					0.73			
**FV3**					0.78			
**FV4**					0.77			
**FV5**					0.25			
**SS1**						0.71		
**SS2**						0.72		
**SS3**						0.81		
**ECOS1**							0.71	
**ECOS2**							0.82	
**ES1**								0.76
**ES2**								0.66
**ES3**								0.82
**ES4**								0.73
**ES5**								0.77

*Internal consistency*. This step assesses the internal consistency reliability, using Jöreskog [88] composite reliability. Higher values indicate higher levels of reliability. The values 0.70 and 0.90 range from "satisfactory to good". Cronbach [89] is another internal consistency reliability test that uses similar thresholds but yields lower results than composite reliability. Because the items are unweighted, Cronbach’s alpha is a less precise measure of reliability. On the other hand, composite reliability weights the items based on the individual loadings of the construct indicators, and reliability is higher than Cronbach’s alpha [84]. Table 4 shows that the values of composite reliability for every construct is greater than 0.70. As a result, all of our constructs meet this requirement.

**Table 4:** Composite reliability and convergent validity.

Variables	Composite Reliability	Average Variance Extracted (AVE)
**Social Mission**	0.7947	0.4949
**Innovation**	0.8083	0.5161
**Collaborative Networks**	0.8074	0.5135
**Level of Scalability**	0.7447	0.4534
**Financial Viability**	0.7688	0.4235
**Social Sustainability**	0.7898	0.5569
**Economic Sustainability**	0.7377	0.5857
**Environmental Sustainability**	0.8662	0.5655

*Convergent validity*. The next step is to assess the convergent validity of each construct measure. The extent to which a construct converges in order to explain the variance of its items is known as convergent validity [90]. The average variance extracted (AVE) for all items on each construct is the metric used to assess convergent validity. To compute the AVE, one must square each indicator’s loading on a construct and compute the mean value [86]. The AVE must be 0.50 or higher to be considered acceptable; an AVE of 0.50 or higher indicates that the construct explains 50 percent or more of the variance in the items that make up the construct [91].

Table 4 presents the values of AVE of all constructs. It can be seen from the table that the values of the three constructs are less than 0.5. However, if the value of composite reliability is greater than 0.6, [92] argue that even if the value of AVE is less than 0.5, it can still be accepted. As a result, all of the values satisfy the criteria.

*Discriminant validity*. The next step is to determine discriminant validity, or how distinct a construct is empirically from other constructs in the structural model [84]. The traditional metric proposed by [92] suggested that each construct’s AVE be compared to the squared inter-construct correlation (a measure of shared variance) of that construct and all other reflectively assessed constructs in the structural model, with the shared variance for all model constructs not exceeding their AVEs [93]. Hulland [87] proposes a correlation matrix that incorporates the square roots of the average variance extracted values determined for each of the constructs along the diagonal and the correlations between distinct constructs in the matrix’s lower left off-diagonal parts.

Table 5 shows that the diagonal elements in the respective rows and columns are greater than the off-diagonal elements, as suggested by [87]. Therefore, discriminant validity has been established for every construct.

**Table 5:** Discriminant validity.

Constructs	Social Mission	Innovation	Collaborative Networks	Level of Scalability	Financial Viability	Social Sustainability	Economic Sustainability	Environmental Sustainability
**Social Mission**	**0.7036**							
**Innovation**	0.5499	**0.7184**						
**Collaborative Networks**	0.5288	0.7153	**0.7164**					
**Level of Scalability**	0.3628	0.5786	0.5434	**0.6722**				
**Financial Viability**	0.3514	0.5717	0.6058	0.6132	**0.6508**			
**Social Sustainability**	0.4202	0.514	0.5441	0.4702	0.4413	**0.7462**		
**Economic Sustainability**	0.3912	0.582	0.5722	0.4797	0.6093	0.4843	**0.7652**	
**Environmental Sustainability**	0.4687	0.65	0.6453	0.544	0.6289	0.4746	0.7087	**0.7519**

#### Assessing structural models

*Variance Inflation Factor (VIF)*. The first step in evaluating structural relationships is to check for collinearity to ensure that the regression results are unbiased [84]. The variance inflation factor (VIF) assesses the indicators’ collinearity [90]. The level of collinearity is greater when VIF values are higher. Collinearity issues between the predictor constructs are indicated by VIF values of 5 or higher. On the other hand, Collinearity issues can occur at lower VIF values of 3 [94,95]. The VIF values should ideally be around 3 and below.

The VIF values are less than 3 (Table 6), indicating no issues with collinearity between the constructs.

**Table 6:** VIF values.

Constructs	Social Sustainability	Economic Sustainability	Environmental Sustainability
**Social Mission**	1.4950	1.4950	1.4950
**Innovation**	2.8719	2.8719	2.8719
**Collaborative Networks**	2.8180	2.8180	2.8180
**Level of Scalability**	1.8392	1.8392	1.8392
**Financial Viability**	1.9469	1.9469	1.9469

*Coefficient of Determination (R*^*2*^*)*. Because collinearity isn’t an issue, in this case, the next step is to look at the endogenous construct’s R^**2**^ value (s). The R^**2**^ is a measure of the model’s explanatory power because it measures the variance that is explained in each of the endogenous constructs [96]. R^**2**^ is a measure of explanatory power that ranges from 0 to 1, with higher values indicating greater explanatory power. R^**2**^ values greater than or equal to 0.75, 0.50, and 0.25 are considered substantial, moderate, and weak, respectively [84]. The adjusted R^**2**^ values are helpful in comparing different models or the model’s explanatory power across different data sets because they consider model complexity and sample size [91].

Table 7 presents the coefficient of determination values. The R^2^ values of social, economic and environmental sustainability are within the acceptable range. The five competencies of social enterprises explain around 36.39%, 46.45% and 55.26% variation in social sustainability, economic sustainability and environmental sustainability respectively.

**Table 7:** Coefficient of determination (R^2^).

Constructs	R Square	R Square Adjusted
**Social Sustainability**	0.3639	0.3532
**Economic Sustainability**	0.4645	0.4555
**Environmental Sustainability**	0.5526	0.5451

*Effect size (f*^*2*^*)*. After calculating R^2^, effect size *f*^2^ is calculated. When a specific exogenous construct is removed from the model, the change in the R^2^ value can be used to determine whether the omitted construct has a significant impact on the endogenous constructs [85]. Small, medium and large effects of the independent variable on the dependent variable are represented by *f*^2^ values of 0.02, 0.15, and 0.35, respectively [97].

Table 8 presents the values of Effect size *(f*^*2*^*)*. For economic sustainability financial viability has a medium effect followed by innovation, collaborative networks, social mission and level of scalability. Environmental sustainability has a medium effect of financial viability, followed by innovation, collaborative networks, social mission and level of scalability. As compared to the other variables, collaborative networks has the maximum effect on social sustainability, followed by level of scalability, social mission, innovation and financial viability.

**Table 8:** Effect size *f*^2^.

Constructs	Social Sustainability	Economic Sustainability	Environmental Sustainability
**Social Mission**	0.0300	0.0223	0.0285
**Innovation**	0.0251	0.0509	0.0760
**Collaborative Networks**	0.1346	0.0278	0.0300
**Level of Scalability**	0.0304	0.0201	0.0207
**Financial Viability**	0.0241	0.2532	0.1538

*Path coefficients (hypotheses testing)*. To fully comprehend the structural model, it is necessary to understand the relevance and significance of the relationships between different exogenous and endogenous constructs. The total effect is the sum of all "direct" and "indirect" effects that link two constructs [98]. The hypotheses are tested using 5000 sub-samples in the bootstrapping process of SMART-PLS [99].

**Fig 6:**
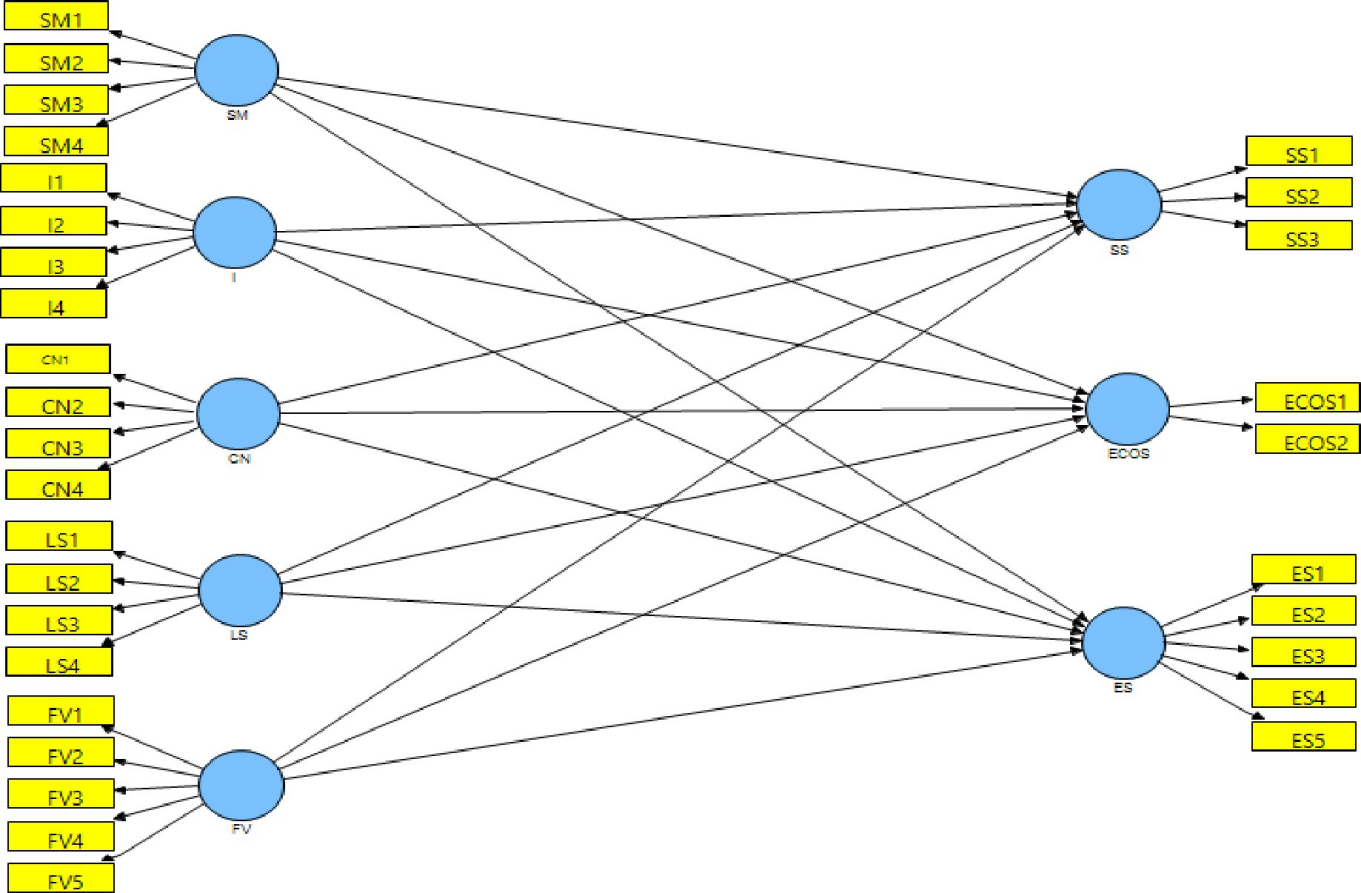
PLS path model. (**Source**: Authors’ Contribution).

Fig 6 shows the PLS path model with all the hypothesized relationships.

Table 9 shows the total effects of each exogenous construct on each endogenous construct, as well as their significance (at the five percent level). The t-statistics values are significant for every relationship. As the table suggests, all the relationships are positive and significant.

**Table 9:** Path coefficients (hypotheses testing).

	Original Sample	T Statistics	Supported/ Not Supported
**Social Mission -> Social Sustainability**	0.1428	7.4943	Supported
**Social Mission -> Economic Sustainability**	0.0598	4.3889	Supported
**Social Mission -> Environmental Sustainability**	0.1094	8.7466	Supported
**Innovation -> Social Sustainability**	0.0944	4.4195	Supported
**Innovation -> Economic Sustainability**	0.2183	9.6732	Supported
**Innovation -> Environmental Sustainability**	0.2258	11.5628	Supported
**Collaborative Networks -> Social Sustainability**	0.2538	11.5793	Supported
**Collaborative Networks -> Economic Sustainability**	0.1353	6.1752	Supported
**Collaborative Networks -> Environmental Sustainability**	0.1872	8.5963	Supported
**Level of Scalability -> Social Sustainability**	0.1817	7.8666	Supported
**Level of Scalability -> Economic Sustainability**	0.0389	2.4326	Supported
**Level of Scalability -> Environmental Sustainability**	0.0938	5.6086	Supported
**Financial Viability -> Social Sustainability**	0.072	3.4448	Supported
**Financial Viability -> Economic Sustainability**	0.3577	21.7053	Supported
**Financial Viability -> Environmental Sustainability**	0.2905	22.7306	Supported

*Predictive relevance*. The Q^2^ value is used to determine the predictive accuracy of the PLS path model [100]. This measure is based on the blindfolding technique, which eliminates individual points from a data matrix, substitutes the mean for the removed points, and estimates model parameters [101]. For a specific endogenous construct, Q^2^ values greater than zero indicate the structural model’s predictive accuracy for that construct [84].

The values of Q^2^ are shown in Table 10. The values of Q^2^ are larger than zero indicating the path model’s predictive relevance for all the constructs.

**Table 10:** Predictive relevance.

Constructs	Q Square
**Social Sustainability**	0.189
**Economic Sustainability**	0.243
**Environmental Sustainability**	0.299

**Fig 7:**
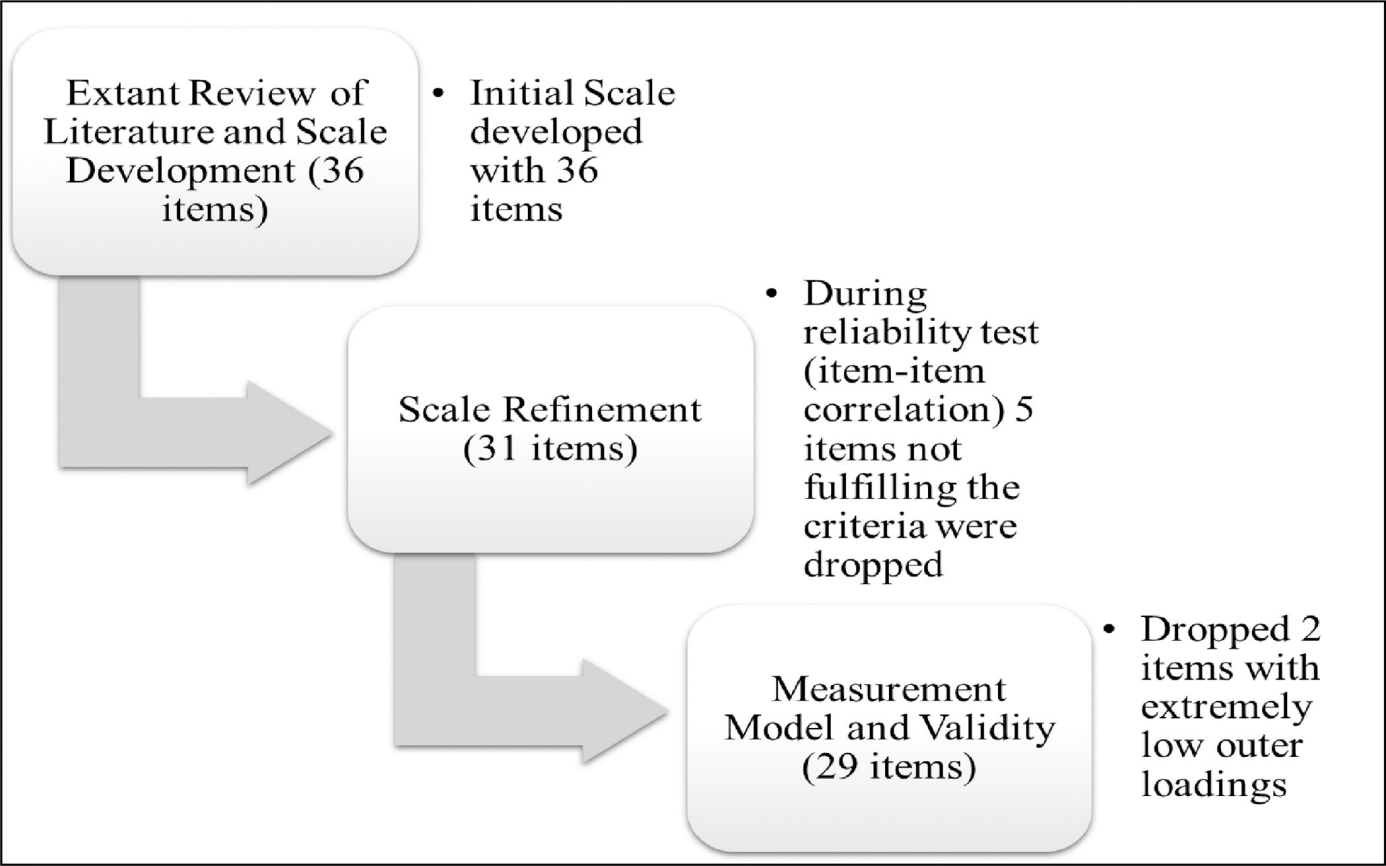
Item reduction procedure. (**Source**: Authors’ Contribution).

Fig 7 depicts the procedure for reducing the overall number of items in each of the three studies. The total number of items is reduced from 36 to 29.

*Standardized Root Mean Square Residual (SRMR) and Normed Fit Index (NFI)*. The standardized root mean square residual (SRMR) is the approximate model fit criterion used in PLS path modelling [91]. The SRMR is the square root of the sum of squared differences between the model-implied and empirical correlation matrices, i.e. the Euclidean distance between the two matrices. A SRMR value of 0 implies a perfect fit, whereas an SRMR value of less than 0.1 indicates an adequate fit [102]. The Normed Fit Index (NFI) examines the difference in chi-squared values between the hypothesised model and the null model. NFI levels greater than 0.90 are considered acceptable [91].

The model fit is good as it is at the recommended value as shown in Table 11 [102].

**Table 11:** Model fit index.

	Saturated Model	Estimated Model
**SRMR**	0.0898	0.0918
**Normed Fit Index (NFI)**	0.9521	0.9394

#### Overall impact of social enterprises on sustainable development

**Fig 8:**
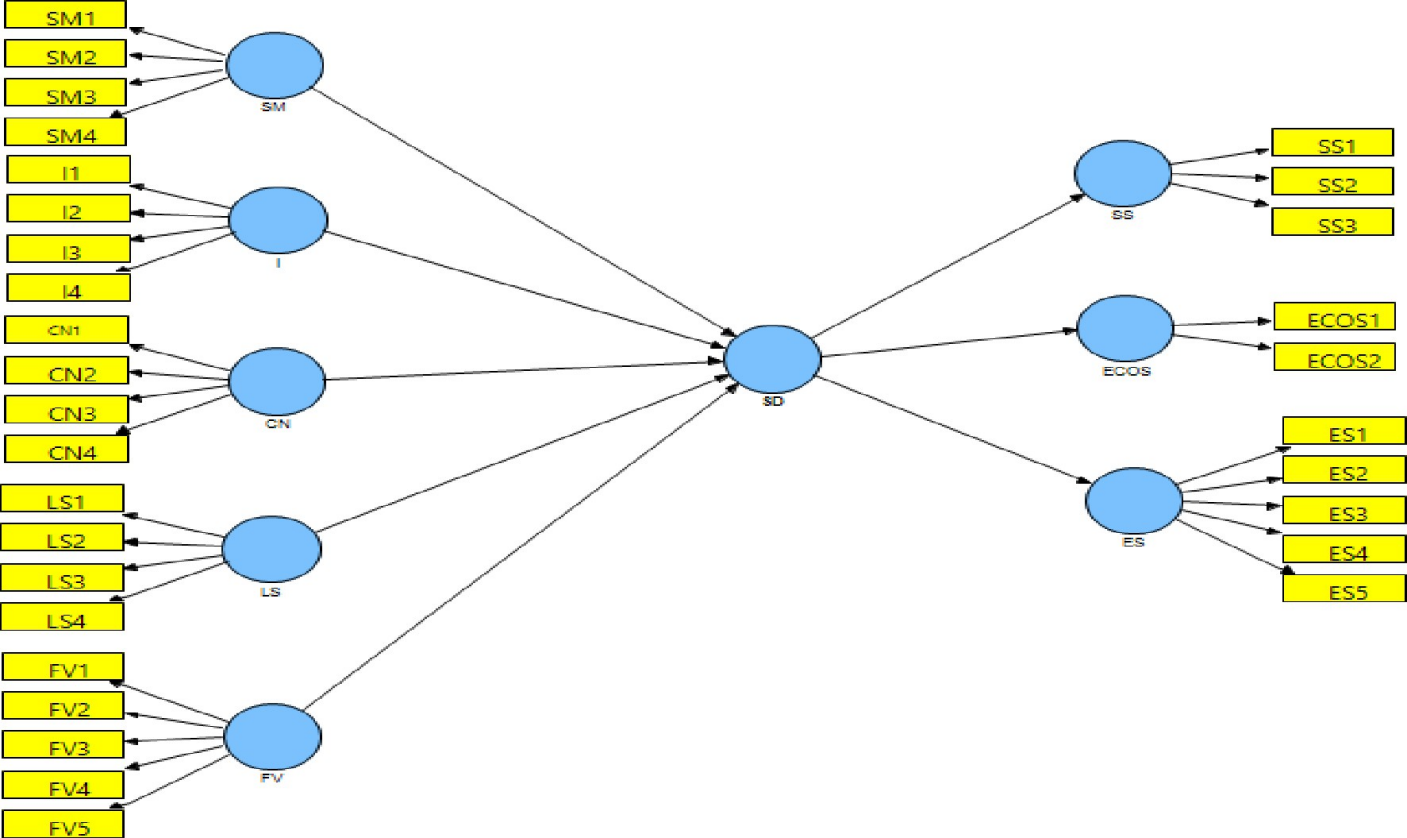
Second order PLS path model. (**Source**: Authors’ Contribution).

**Fig 9:**
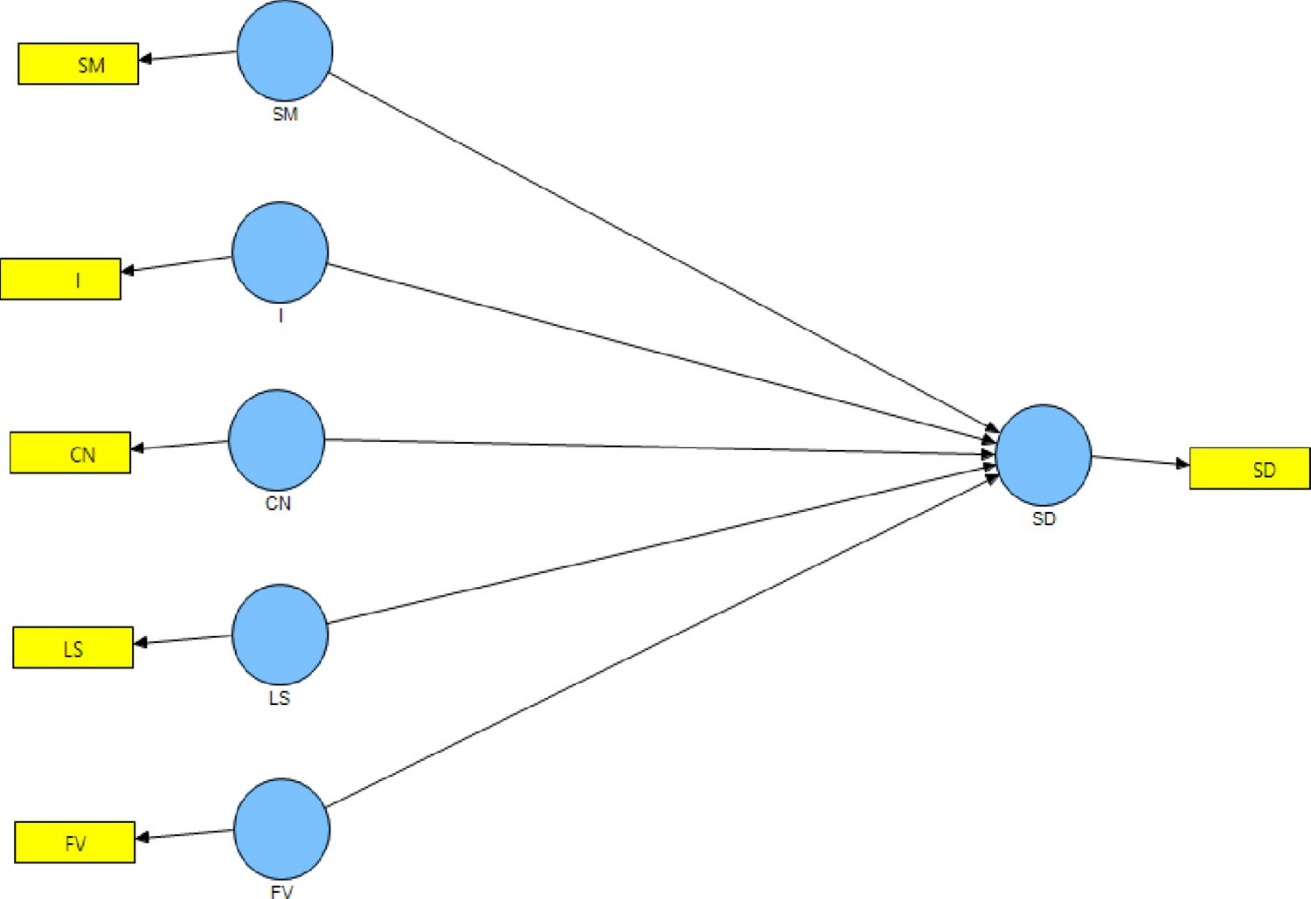
First order PLS path model. (**Source**: Authors’ Contribution).

The analysis presents the impact of five competencies of social enterprises on sustainable development. To assess the structural model, the 2^nd^ order model (Fig 8) is condensed into 1^st^ order (Fig 9) using the latent variable scores (LVS) obtained through the analysis of the original model [103].

Table 12 shows that sustainable development has a significant R^2^ value of 0.642, i.e. the five competencies of social enterprises explain around 64.2% variation in sustainable development.

**Table 12:** Coefficient of Determination (R^2^).

	R Square	R Square Adjusted
**Sustainable Development**	0.642	0.641

*Predictive relevance*. The value of Q^2^ is greater than zero as shown in Table 13, indicating the structural model’s predictive accuracy for sustainable development.

**Table 13:** Predictive relevance.

Constructs	Q Square
**Sustainable Development**	0.627

*Effect size*. Table 14 shows that collaborative networks, financial viability and innovation have significant impact on sustainable development with *f*^2^ value of 0.462, 0.753 and 0.430 respectively. Level of scalability (0.327) and social mission (0.345) have medium effect on sustainable development.

**Table 14:** Effect size.

	Sustainable Development
**Social Mission**	0.345
**Innovation**	0.430
**Collaborative Networks**	0.462
**Level of Scalability**	0.327
**Financial Viability**	0.753

*Path coefficient*. Table 15 presents that social mission, innovation, collaborative networks, level of scalability, and financial viability have a significant positive impact on sustainable development at 5% significance level.

**Table 15:** Path coefficients.

	Original Sample	T Statistics	P Values
**Social Mission -> Sustainable Development**	0.173	9.271	**0.000**
**Innovation -> Sustainable Development**	0.270	9.376	**0.000**
**Collaborative Networks -> Sustainable Development**	0.275	8.920	**0.000**
**Level of Scalability -> Sustainable Development**	0.188	8.175	**0.000**
**Financial Viability -> Sustainable Development**	0.292	10.571	**0.000**

## Discussion

The social enterprise movement is emerging as a long-term solution for persistent societal issues that markets and governments have been unable to address. These societal problems are common around the globe and have been attributed to inequalities, market and government failures that are depicted by marginalization and social exclusion [33]. The social enterprise ecosystem provides numerous opportunities to connect with local partners, learn, and pursue innovative solutions to multiple social challenges in healthcare, agriculture, education, manufacturing, renewable energy, and skills development [104].

The study’s theoretical framework explains how various aspects of social enterprises (social mission, innovation, collaborative networks, Level of Scalability, and financial viability) contribute to sustainable development. Because the agendas of social enterprises and sustainable development are complementary and mutually reinforcing, the study concludes that promoting social enterprises may aid in achieving the goal of sustainable development. The integrative review presented in the study helps researchers identify the current state of research in the field and highlights future research directions.

All the hypothesized relationships have been supported by the literature. The results of the path coefficient suggest that social mission impacts social, economic, and environmental sustainability. Also, from the extant literature, the purpose and goal of establishing a social enterprise are defined by the social mission [105]. Doherty et al. [106] explain that market forces cannot divert social entrepreneurs from their mission due to their high commitment. Social enterprises provide positive socioeconomic value to communities by working tirelessly to find long-term solutions to social issues. Furthermore, during an environmental disaster, the social purpose provides an excellent technique for connecting with external people while keeping internal people safe [107]. The social mission gives a clear path for achieving long-term development while minimising environmental damage. Therefore, social mission aids individuals from dangerous situations, increases community growth, and protects the environment [108].

Innovation positively impacts social, economic as well as environmental sustainability. Social innovation is developing a new and practical response to a social issue that adds value to society [109]. To find sustainable solutions to societal issues that governments or commercial sectors have disregarded, social entrepreneurs create a synergistic combination of products, capabilities, procedures, and technology [110]. In this way, social innovation aids entrepreneurs in achieving long-term success. Social entrepreneurship is a promising tool for commercial and social value creation [111]. It is also tied to the process of product, service, and technological innovation to address social requirements in various environmental problems [61]. Social innovation contributes to sustainable growth and meets social demands while preserving the environment [112].

The results suggest that collaborative networks positively impact social, economic, and environmental sustainability and are supported by the literature [34,67,113]. Collaborative networks are groups of people and organisations that are linked together and share their knowledge and resources [67]. Shareholders participate in influencing organisations’ social and environmental responsibilities through the practice of socially responsible investment. Firms are forming partnerships in the form of networks to deal with uncertainty and unexpected environmental changes. Entrepreneurs can use social networks to find opportunities to access resources and information, establish connections with people and society, share knowledge, and close the asymmetry gap between different stakeholder groups [113].

The level of scalability positively impacts sustainable development. The result has been supported by many researchers, including [34,59,114]. As business entrepreneurs build businesses that produce goods or render services and make money, social entrepreneurs build and manage organisations that do the same. These enterprises aim to benefit society and the environment while also, to some extent, earning profit. Creating social value and making a profit are not mutually exclusive in a social enterprise. The challenge is ensuring that a sufficient mix of measurements is employed to capture the social enterprise’s varied outputs. Firm growth can take many forms, not all of which must occur simultaneously. For example, in the early stages of a social enterprise’s life, change typically occurs in non-financial areas. It then progresses to the potential to grow in revenue (profit), employment, and product/service growth as the firm matures.

The results of the path coefficient have supported the hypothesised relationship between financial viability and sustainable development. Social entrepreneurs aim to create total wealth [63], representing both social value and economic wealth for the enterprise to survive. As a result, according to [115], social entrepreneurs must satisfy their investors by providing a Return On Investment (ROI) while also being socially effective by maximising social ROI. However, due to a lack of funding, many organisations transit to commercial operations to produce the necessary financial resources [13]. These organisations must change from the concept of "cost recovery" to "more than cost recovery" to attain sustainability and become self-sustaining social organisations [12]. Parnell [116] argues that businesses who use sustainable environmental practises rather than polluting the environment gain more advantages and stay competitive. Social enterprises and the natural environment are also intertwined. Social enterprises seek social and commercial benefits while adhering to environmental sustainability principles. Social companies follow policies that promote environmental sustainability, such as eco-efficiency, eco-equity, and eco-effectiveness.

## Conclusion

This study adds to the existing literature on social entrepreneurship by elucidating the impact of these businesses. To the best of our knowledge, this is the first-ever study to test how social enterprises help achieve sustainable development empirically. The study significantly contributes to the extant literature in four ways. First, the paper proposes a conceptual framework to study the relationship between social enterprises and sustainable development. Second, it empirically tests the framework using a structured questionnaire. Using PLS-SEM, the paper analyzed 305 responses collected from social entrepreneurs. The findings suggest that the competencies of social enterprises (that is, social mission, innovation, Level of Scalability, financial viability and collaborative networks) have a significant impact on sustainable development in the country. Third, it has significant implications for India, a developing country that other countries can replicate. By putting the proposed framework to the test in that country, similar implications for other developing countries could be drawn. Fourth, the integrative review presented in the paper sheds light on the current state of literature and presents future research avenues.

## Implications and limitations of the study

### Implications for social entrepreneurs

- The findings can motivate social entrepreneurs to use social innovation and collaborative networks to make a greater positive effect. Furthermore, this shall put competitive pressure on other businesses to adopt creative and sustainable management practices, resulting in a more sustainable society.
- Establish a system of networking for social and commercial enterprises. Both kinds of entrepreneurs will be able to exchange expertise through this network. The social entrepreneur can help the commercial entrepreneur to put more emphasis on the company’s social mission. On the other hand, the commercial entrepreneur could assist the social entrepreneur by applying pertinent commercial insight to the social enterprise.
- Creating a panel of mentors for social entrepreneurs can be a great resource for offering guidance, knowledge, and workable answers to the social entrepreneur in handling the interpersonal and professional disputes that arise during the expansion of the social enterprise.
- Social networking among businesses increases the market worth of social entrepreneurs and allows them to impact social innovation.
- Social entrepreneurs drive market competition through social missions, cutting-edge environments, and economic growth strategies.
- Social entrepreneurs can interact with customers, rivals, and other stakeholders to assist them with setting and managing prices and to lessen customers’ price sensitivity
- Social entrepreneurs who take the initiative deliberately involve and engage their customers and create new value propositions with them. Building a new product with ongoing customer feedback can foster a sense of shared ownership and secure the customers’ pre-commitment, which reduces the possibility of loss and enhances the likelihood of attracting more customers.

### Implications for policymakers

- Policymakers can make policies and programs to support and promote existing and potential enterprises and run awareness camps to make their contribution visible to the general public.
- Policymakers can directly assist social entrepreneurs by providing numerous opportunities in terms of government funding. However, to create more social entrepreneurs and support existing ones, we must learn more about the pattern of company growth and the challenges faced in creating the social enterprise.
- The Policymakers can make social enterprises eligible to use the current schemes or policies deployed for them to help the social enterprise move away from the struggle to validate their organization status in terms of revenue or growth before they start using policy to benefit the users.
- Business skill development in the social entrepreneurship sector in developing nations like India, needs to be promoted. Most social enterprises in India are created through non-governmental organizations or unofficial channels.
- Presently, various measures for entrepreneurs help them grow their businesses. However, most social entrepreneurs are not aware of such measures. It might be because the existing measures are not that stringent and effective.
- The governments may provide social entrepreneurship a major push by financing and facilitating institutions of higher learning that are specifically dedicated to advancing entrepreneurial teaching and research.

### Limitations of the study

The study, like any research initiative, has some *limitations*. These flaws have left some gaps in the research that can be investigated more in the future. The study is cross-sectional. Future research can use this model on longitudinal design research to more generalize the results. A limited number of social enterprises were reached in the study. A larger sample with a broader range of enterprises from different sectors will shed more light on this topic. There is still much about the engagement of social enterprises with the previous MDGs that we do not know, and indeed it would be interesting to compare this with emerging patterns of engagement with the SDGs.

## Supporting information

S1 File (XLSX)
